# Applying the Alkali-Activation Method to Encapsulate Silicon Nitride Particles in a Bioactive Matrix for Augmented Strength and Bioactivity

**DOI:** 10.3390/ma17020328

**Published:** 2024-01-09

**Authors:** Guido Manuel Olvera de la Torre, Monika Tatarková, Zuzana Netriová, Martin Barlog, Luca Bertolla, Miroslav Hnatko, Gianmarco Taveri

**Affiliations:** 1 Institute of Inorganic Chemistry (ICC), Slovak Academy of Sciences (SAV), Dubrávska cesta 9, SK-845 36 Bratislava, Slovakia; guido.olvera@savba.sk (G.M.O.d.l.T.); monika.tatarkova@savba.sk (M.T.); zuzana.netriova@savba.sk (Z.N.); martin.barlog@savba.sk (M.B.); miroslav.hnatko@savba.sk (M.H.); 2 Institute of Physics of Materials (IPM), Academy of Sciences of the Czech Republic (ASCR), Žižkova 22, 117 20 Brno, Czech Republic; bertolla@ipm.cz; 3 Centre for Advanced Materials and Applications (CEMEA), Slovak Academy of Sciences (SAV), Dubrávska cesta 9, SK-845 36 Bratislava, Slovakia

**Keywords:** bioceramic, silicon nitride, alkali-activation method, combeite, bioactivity

## Abstract

The development of bioactive ceramics still poses challenges in finding a good compromise between bioactivity and mechanical robustness. Moreover, a facile, low-cost and energy-saving synthesis technique is still needed. This study concerns the synthesis of a bioactive material by growing a bioactive Na-Ca-Mg-Si-based ceramic matrix produced using the alkali-activation method on silicon nitride (Si_3_N_4_) particles. This technique simultaneously forms the matrix precursor and functionalizes the Si_3_N_4_ particles’ surface. The optimal strength–bioactivity compromise was found for the composition containing 60 wt.% Si_3_N_4_ and 40 wt.% of the matrix exhibiting good compressive strength of up to 110 MPa and extensive precipitation of hydroxyapatite on the sample surface after 7 days of soaking in simulated body fluid. This innovative approach merging strong non-oxide binary ceramics with the versatile and low-cost alkali-activation method holds great expectations for the future in biomaterials.

## 1. Introduction

Biomaterials [1] are natural or synthetic materials used in various medical applications to support, enhance, or replace damaged tissues or biological functions. They have gained increasing attention due to the aging population [2,3]. Consequently, researchers are striving to find suitable materials for diverse applications. However, challenges such as processing, which play a pivotal role in harnessing these properties, material half-life, and the complex shapes of the human body make it difficult to rely on just one material or shape. 

Bioceramics are typically produced through high-temperature calcination of preformed or purchased precursors. Commonly, precursors are homogenized via ball milling before calcination [4]. In the case of glasses and glass ceramics, the precursor mix is melted and then quenched to obtain a glassy product [4,5,6]. Bioglasses can also be synthesized at room temperature using the sol-gel method, where dissolved silicon alkoxide species hydrolyze to form nanometric silica particles in a colloidal-like solution (“sol”) which eventually cluster up in a 3D framework embedded in the solvent (“gel”) [4,6]. However, this method usually requires expensive and toxic chemicals.

The densification of these bioceramics is achieved through powder sintering, wherein the compacted dry powder (green body) is fired up to 2/3 of its melting temperature (T_m_), often exceeding 1000 °C. To address environmental concerns and promote eco-friendly practices, alternative methods are being explored, such as alkali activation [7,8], forming alkali-activated materials (AAMs). In this method, an alkaline solution is used to partially dissolve the powder and precipitate of an amorphous substance (usually alkali-silicate) on the surface of an unreacted fraction. This substance hardens upon water evaporation, similarly to cement [9]. Alkali cations (usually Na and K) modify the silica network to form a hydrated alkali-silicate phase (N-S-H), whereas in cementitious materials, the reaction product is typically a hydrated calcium-silicate compound (C-S-H). One significant advantage of this method is the possibility to recycle silica-based industrial wastes, minimizing production costs [10]. Despite its similarities to types of cement, AAMs have been widely employed in construction but neglected in biomedicine. Although self-setting biomaterials based on different technologies have been produced, their results remain decent but insufficient [11].

Silicon nitride (Si_3_N_4_) is also considered a kind of biomaterial. Firstly synthesized by Deville and Wöhler in 1857 [12], initially, it was meant to be refractory material [13] due to its thermal properties and excellent mechanical strength, including high flexural strength (800–1100 MPa) [12] and fracture toughness (~5 MPa·m^1/2^) [14], making it suitable for applications such as rocket nozzles, thermocouple tubes, and crucibles for molten systems. Since 2017, Si_3_N_4_ has gained popularity in the field of biomaterials [15,16] due to its exceptional mechanical properties as well as its biocompatibility [17,18] and antibacterial properties [19,20]. It has found preliminary applications in orthopedics [21] and dentistry [22]. However, obtaining dense silicon nitride samples has traditionally required extreme sintering temperatures, and less energetically costly techniques like hot pressing [23,24], hot isostatic pressing [25,26,27] or spark plasma sintering [28,29,30] often lead to extreme contamination. Notoriously, all the silicon-based binary non-oxide hard ceramics, including Si_3_N_4_, develop a nanometric passivated layer of SiO_2_ on the particles’ surface, which can be easily functionalized through alkali activation. In a typical corrosion cycle, this oxide layer can be easily dissolved in an alkali-base solution and a new passivated layer is then formed. The surface corrosion of Si_3_N_4_ balls in a diluted LiOH solution was studied and it was found to exhibit Arrhenius’ behavior with respect to temperature [31].

In this work, we will elucidate the possibility of applying the alkali-activation method to produce a bioactive material that strongly bonds to Si_3_N_4_ particles by functionalization of their surface oxide layer. The prospect of generating a new generation of biocements through the alkali activation of biocompatible hard ceramics (such as Si_3_N_4_) may mark a groundbreaking shift in healthcare. This approach enables the synthesis of dense materials using a single cost-effective and eco-friendly fabrication method. We believe that this innovative approach to biomaterials synthesis will open up new avenues for designing and engineering bioimplants and prostheses.

## 2. Materials and Methods

The chemicals used for the synthesis of the AAMs are the following: Si_3_N_4_ (grade SN-E10, >95% α-Si_3_N_4_, d_50_ = 0.5 µm, O < 2.0 wt%, UBE Corporation, Tokio, Japan) to disperse in the matrix composed of magnesium oxide (MgO, purity 97%, Merck, Darmstadt, Germany), silicon dioxide (SiO_2_, Aerosil OX-50, Evonik Industries. Essen, Germany), and calcium hydroxide Ca(OH)_2_. The alkaline activator is a sodium hydroxide solution created by dissolving NaOH pellets (purity 98%, Merck, Darmstadt, Germany) in distilled water. This proportion was used to mimic the bioglass composition for optimal bioactivity of the matrix [32]. The silicon nitride-to-matrix ratio employed in the experimental plan of this study is shown in Table 1. In a typical experiment, the powder mixture was initially homogenized in a planetary ball mill (model PM100, Retsch GmbH, Haan, Germany) for 30 min at a rotation speed of 250 rpm, using isopropanol as the slurry medium. Subsequently, the suspension was dried using a rotary evaporator. The homogenized powder was already mixed with our alkaline activator. It must be pointed out that the saturation limit of NaOH in a water solution is 23-25 M, thus the alkali activator for the SI40 and SI50 compositions is already a supersaturated solution. The resulting slurry was then poured into a cylindrical plastic vial. To remove air bubbles, the slurry was cast into the container and manually vibrated. The vials were sealed and the pastes were cured in an oven at 80 °C for 24 h to allow a complete reaction, followed by an additional 24 h with open vials to evaporate the water. Subsequently, the samples were calcinated at a temperature of 750 °C in the air atmosphere using a Clasic 0213 T furnace equipped with a Clasic Clare 4.0 temperature controller (Clasic CZ s.r.o., Revnice, Czechia). Both the heating and cooling rates were set at 10 °C/min, and the samples were held at the maximum temperature for 3 h in an alumina crucible. The density of the samples was measured using the Archimedes method, where 10 mm disks approx. 5 mm thick were weighted using an analytical balance (Kern ABT 120-4M, Kern Ltd., Frankfurt am Main, Germany) with an accuracy of up to 0.0001 g, and successively immersed in ultra-pure water and let rest for 5 min before measuring the wet mass of the same. The measurement was repeated 5 times for each sample and the average was taken for calculation. The relative density was then calculated with respect to the theoretical density of the material which varies according to the composition of the samples (i.e., the Si_3_N_4_ to matrix ratio). The theoretical density of the matrix was that of a combeite phase. 

The infrared spectra of the samples were obtained using a Fourier transform infrared (FTIR) spectrometer Nicolet 6700 from (Thermo Fisher Scientific, Waltham, MA, USA) equipped with an IR source, KBr beamsplitter, and DTGS detector for the middle-IR (MIR) region (4000–400 cm^−1^). The spectra were measured in the MIR region using the KBr pressed disk technique (approx. 1 mg of sample and 200 mg of KBr was used) with 64 sample scans and a resolution of 4 cm^−1^. The KBr pellets were heated for 24 h at 110 °C to remove most water adsorbed on KBr. Manipulation of the spectra was performed using the OMNIC™ software package (OMNIC 8.3, Thermo Fisher Scientific, Waltham, MA, USA).

The thermal evolution of the alkali-activated samples (SI80, SI60 and SI40) was examined through TGA/DTA thermal analysis up to 1200 °C in the air with a ramp of 10 °C/min, using an STA 449 F3 Jupiter (Netzsch Gerätebau GmbH, Selb, Germany). Disposable alumina crucibles were used for the measurements. This technique permits the determination of the optimal temperature of calcination. 

The compressive strength of cylindrical samples was measured using a universal testing machine (Series Pro line, ZwickRoell GmbH & Co., Ulm, Germany) with a load speed of 0.2 mm/min. The load speed was appropriately chosen to allow a smooth increase of the stress–strain curve. The machine load cell is 250 kN, the precision of the crosshead is ±2 μm and the resolution of the drive system is 0.19227 nm. The application of the test load was controlled according to the DIN EN ISO 7500-1 standard [33]. A minimum of 10 intact specimens suitable for testing were selected from each batch. The shape of the specimens was cylindrical with a diameter of approx. d = 10 mm and height of at least h = 1.5 d to avoid the size effect on compressive strength. The tested calcinated samples were from SI50 to SI70 compositions because no testable specimens could be obtained for the SI40 composition due to crack formation. Non-calcinated SI40, SI50, SI60 and SI70 compositions were also tested to quantify the benefits of the calcination stage in terms of mechanical strength. Additionally, Vickers hardness was evaluated under a load of 9.81 N on both AAMs and calcinated samples. The measurements were conducted on a screwdriven Zwick Z2.5 machine (Zwick Roell GmbH & Co., Ulm, Germany) equipped with a micro-hardness head ZHU0.2. The load cell of the testing machine goes up to 2.5 kN, and the micro-hardness head equipped with instrumented Vickers, Knoop and universal micro-hardness test had a load of up to 200 N and a position resolution of 20 nm. The fracture toughness of the calcinated samples was studied using the indentation fracture method (IF method), after obtaining the Vickers hardness values, and the lengths of cracks for each indentation were measured. Antsis’ formula [34] was then applied to calculate the fracture toughness.

A morphological evaluation of the polished microstructure was conducted on bulk samples using a scanning electron microscope (SEM) (EVO 40 HV, Carl Zeiss, Oberkochen, Germany) to study microstructural homogeneity. Polishing was carried out in EcoMet 30 with diamond suspensions (9 μm → 1 μm) to produce a mirrored finish.

The in vitro bioactivity assay was carried out by immersing samples in simulated body fluid (SBF), prepared according to the Kokubo method [35]. During the soaking process, the SBF was contained in sterile plastic containers, with the amount used for each sample determined by the sample’s total surface area. These containers were sealed and placed in an incubator (Binder Model B28, Binder GmbH, Tuttlingen, Germany) held at a constant temperature of 36.5 °C. The samples were tested at 1, 3, 5, and 7 days. After each soaking period, the samples were washed with pure water and then dried at room temperature in a desiccator. The formation of the apatite layer on the sample surfaces was determined using SEM and EDS. 

## 3. Results

### 3.1. Materials Characterization

**Figure 1:**
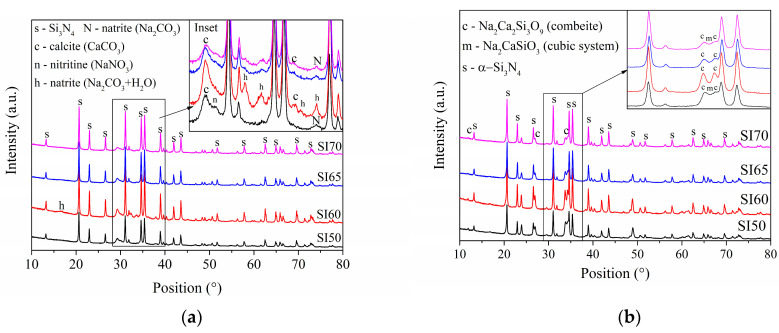
XRD patterns of the (**a**) alkali-activated materials and (**b**) calcinated samples. Insets: magnification of the portions of the XRD patterns to highlight the phases of the precursors and the ceramic forms.

Figure 1 reports the XRD patterns of different samples both for AAMs only (Figure 1a) and calcinated samples (Figure 1b). In Figure 1a, besides the intense diffraction patterns related to α-Si_3_N_4_, the products of alkali activation are also visible, although they are slightly different among compositions, in the region of 25–40° (inset in Figure 1a). In this region, a small halo due to an amorphous phase is present. Furthermore, the calcite phase (CaCO_3_) and the natrite phase (Na_2_CO_3_) were detected in all samples, but the SI50 sample also shows an intense peak related to the nitritine phase (NaNO_3_). In this composition, the concentration of the supersaturated alkaline activator (see Table 1) is such that it degrades the Si_3_N_4_ particles and induces the release of ammonia which, in turn, forms NaNO_3_ while reacting with NaOH. Interestingly, the SI60 sample presents a sizeable amount of hydrated Na_2_CO_3_∙H_2_O phase (called thermonatrite) which becomes less intense in the other compositions with more Si_3_N_4_ content. Already in this sample, the activator is no longer supersaturated and nitritine content is, indeed, considerably less, meaning that Si_3_N_4_ particles are preferentially etched by concentrations close to the saturation point. However, the XRD pattern of the SI70 sample is found to be similar to that of SI50, except for the nitritine content. After calcination (Figure 1b), all the samples produce XRD pattern peaks typical of a hexagonal combeite phase (Na_2_Ca_2_Si_3_O_9_) [36], but the extreme compositions again show similar patterns, by producing, alongside the combeite, a cubic NaCaSiO_2_ phase (see inset in Figure 1b). The Rietveld refinements show consistent values for the SI50 and SI60 samples, i.e., 50–50 wt% and 60–40 wt% of Si_3_N_4_-ceramic matrix, respectively, but are found to be mismatched with the theoretical ones (68–32 wt% and 75–25 wt%, respectively) for the SI65 and SI70, with an increasingly higher gap with less matrix content. Later on, this counterintuitive result will be discussed.

**Figure 2:**
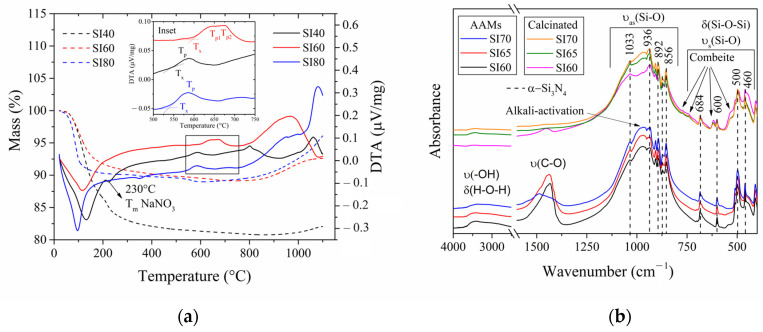
(**a**) TG and DTA curves of the following compositions SI40, SI60 and SI80 (inset: magnification of the DTA exothermic peaks related to the crystallization temperatures), and 2 (**b**) FTIR spectra of alkaline-activated and calcinated samples in the range of 4000 to 450 cm^−1^ interrupted between 2500 and 1600 cm^−1^.

Figure 2a shows the thermal analysis conducted on the AAMs to understand their thermal behavior. The TG measurements indicate that the major mass loss occurs at approximately between 50 °C and 250 °C. This loss is attributed to the presence of free water, and water absorbed in small pores or on the surface of hydroxyl groups [37,38]. Between 250 °C and 700 °C, a minor mass loss of about 2% can be noticed, attributed to the release of silicates’ hydroxyl groups, associated with more tightly bound water related to silanol bonds (Si-O-H) [39]. The SI40 sample has a greater weight loss due to the presence of the highly hygroscopic NaOH in the supersaturated solution. Above 800 °C, a crystallization and its consequent volumetric expansion associated with this range of temperatures occurs [39]. 

The DTA curve shows the occurring chemical reactions and phase transitions during the firing of the samples. From 22 to 40 °C, a small endothermic peak indicates the initial stages of water evaporation. However, until 120 °C, a larger endothermic peak corresponding to complete water evaporation can be observed. This shoulder in peaks can be attributed to the two water evaporation modes explained above. This contribution is similar in all the samples, though occurring at higher temperatures with increasing matrix content, again due to hygroscopic NaOH. The SI40 composition possesses an endothermic peak at 230 °C related to the melting temperature of NaNO_3_, found to be prominent in the XRD patterns of the alkali-activated SI50 sample (Figure 1a), confirming it to be an effect of the supersaturated NaOH solution. An endothermic peak at 350 °C can be distinguished in the SI80 composition, though the source of it is uncertain. The formation of combeite after the process of recrystallization in a general bioglass is characterized by an endothermic peak associated with a temperature of glass transition (T_g_) around 550 °C, along with high exothermic peaks related to the recrystallization temperatures (T_x_,T_p_) between 700–800 °C [40]. The former is absent because the precursor is not glass but an AAM, but the latter is evident in all the compositions. Indeed, the crystallization of the alkali-activated precursors follows more a solid–state reaction rather than a bioglass recrystallization: the advantage of the alkali-activation method over a solid–state reaction is high homogeneity of the precursors, inducing a fast calcination reaction. The SI60 composition shows an onset of the crystallization (T_x_ = 600 °C) and two crystallization peaks (T_p1_ = 645 °C and T_p2_ = 675 °C, see inset in Figure 2a) comparable to Zandi’s work [41]. However, the extreme compositions showed a different trend (T_x_ = 550 °C and T_p_ = 584 °C). This is due to a calcination reaction following the formation of the cubic NaCaSiO_2_ phase (see Figure 1b), which results from the chemistry of alkali-activated precursors (see Figure 1a). Moreover, the SI40 composition shows another exothermic peak at 800 °C of unclear origin. Finally, above 800 °C, strong exothermic peaks are related to the onset of melting. 

FTIR spectra (Figure 2b) were obtained for the AAMs and calcinated samples. Figure 2b focuses on the FTIR spectra from 450 to 1250 cm^−1^, which are related to silicon elements and their respective bands. The peaks from 420 to 690 cm^−1^ are associated with Si-O-Si bending vibration [10,42] and symmetric stretching of the silicate network [37], whereas the large band between 800 and 1200 cm^−1^ is associated with the Si-O asymmetric stretching vibration [9]. Most of these peaks, marked with dashed lines in Figure 2b, are found in both AAMs and calcinated materials, as they are characteristic peaks of Si_3_N_4_ [43]. In particular, the peak at 460 cm^−1^ is in common with a product of alkali-activated silica. However, the main trait of the alkali activation is an amorphous hump between 900 and 1100 cm^−1^ [9]. In the calcinated samples, the formation of the ceramic combeite matrix is characterized by a dwindling of this hump and the appearance of four small peaks at 523, 620, 775 and 790 cm^−1^ [40]. Moreover, a large peak around 1400–1500 cm^−1^ related to the stretching mode of O-C-O elements is found only in the AAMs, a typical sign of atmospheric carbonation [9] (determined by the calcite and sodium carbonate phases detected in the XRD patterns). This peak is not present in the calcinated samples, or to an extent only in the SI60, perhaps due to an incomplete crystallization reaction or adventitious contamination. Finally, a large hump between 2500 and 3500 cm^−1^ related to the stretching vibration mode of –OH and bending mode of H-O-H elements indicates the presence of chemical water and hydrated compounds in the AAMs only [9].

### 3.2. Mechanical Tests 

**Figure 3:**
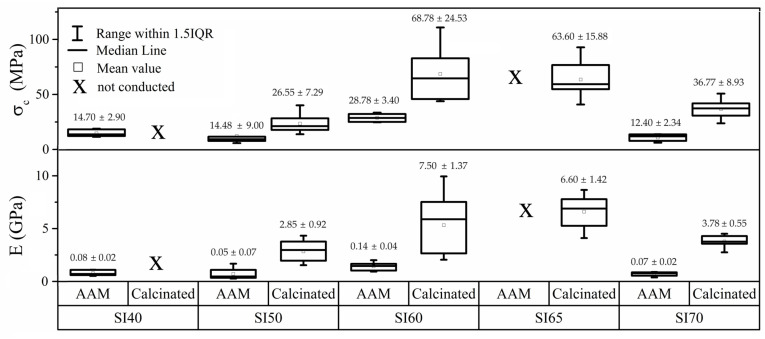
Compressive strength (**upper plot**) and elastic modulus (**lower plot**) of alkali-activated and calcinated samples.

**Figure 4:**
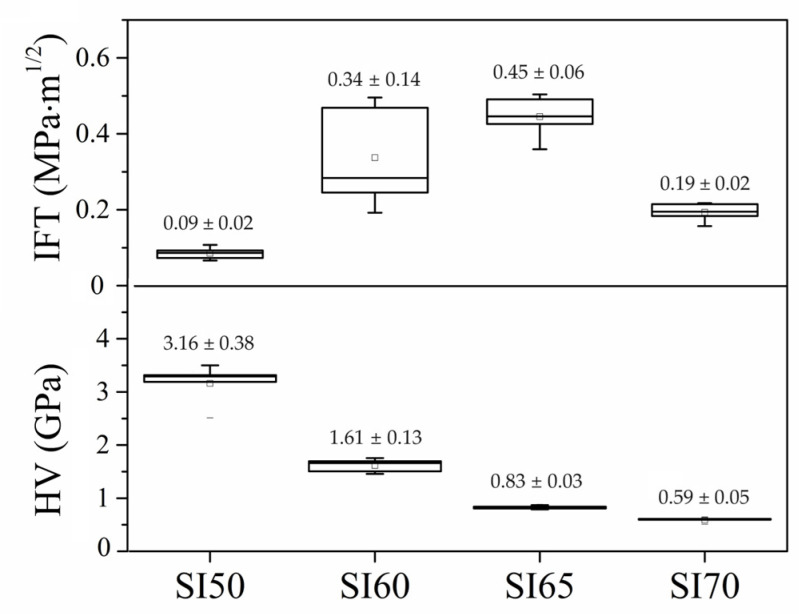
Values of the micro-indentation hardness (**lower plot**) and indentation fracture toughness (**upper plot**) of the calcinated samples.

The choice to conduct mechanical tests on AAMs was only made to provide a comparison with the calcinated materials in an attempt to quantify with sheer numbers the extent of mechanical improvement upon calcination. Indeed, the calcination stage is an essential step without which the bioactive additives in a composition cannot be functionalized. As shown in Figure 3, the compression strength of the samples was measured both before and after calcination. The graph indicates a trend where a higher matrix content (SI40 and SI50) leads to a rather low compressive strength (σ_c_), with values in a range from 10 to 15 MPa. However, samples with 60% silicon nitride (SI60) exhibit significantly better compressive strength, on average 26.55 ± 7.29 MPa. Nevertheless, when the silicon nitride content increases to 70%, the strength begins to decrease, reaching values similar to those observed at 40%. Moreover, the statistic provides a very narrow dispersion. All the AAMs are found to exhibit ductile properties due to the hydrated alkali-activated matrix, as exposed in the elastic modulus values (E) in Figure 3. The SI80 sample has no relevant mechanical properties and is therefore omitted from the experimental plan. For the same reason, the calcinated SI80 sample is omitted, whereas the SI40 sample exhibits a large formation of cracks which impedes the production of testable samples (see Figure 4). Formation of cracks is commonly observed in AAMs, and especially after the process of calcination due to water evaporation. The compressive strength values of all the calcinated samples are found to be optimal again for the samples SI60 and SI65, that is 68.78 ± 24.53 MPa and 63.60 ± 6.60 MPa on average, respectively. This increase can be attributed to the formation of the glass-ceramic phase, identified as combeite. It must be pointed out that also in these samples, high scattering of the data was due to the formation of cracks after calcination. Indeed, the SI60, with the largest data dispersion (see Figure 4), also shows the highest max strength of 110 MPa. The formation of cracks, therefore, veils the real performance of the material. This very well may apply to the SI50 sample, which exhibits lower strength and narrower scattering, as the formation of cracks increases with the matrix content. The SI65 composition was only tested for calcinated samples to give a hint of the declining trend of the strength. Indeed, with 70% of Si_3_N_4_, the average σ_c_ decreases significantly to 36 MPa with a decisively higher ductility, most likely due to the presence of unreacted products. The values of E in this set of samples are calculated to be in all the cases way higher than the AAMs, a characteristic of strong glass ceramics. The micro indentations conducted on the calcinated samples offer a better perspective on the real potentiality of the material, because the test is less affected by cracks formation as the spot may be conveniently selected. The SI50 shows, indeed, a decisively high value of hardness, 3.16 ± 0.38 GPa, with the same property declining with increasing amounts of silicon nitride (see Figure 4). The SI50 sample has a slightly larger scattering compared to the others because the extensive formation of cracks affects the micro-indentation test more. The respective values of indentation fracture toughness (IFT) calculated according to the Anstis theory [34] are reported in Figure 4, and again show a trend comparable to the compressive strength, because the formation of the Palmqvist cracks is, on the contrary, highly affected by surrounding defects. 

**Figure 5:**
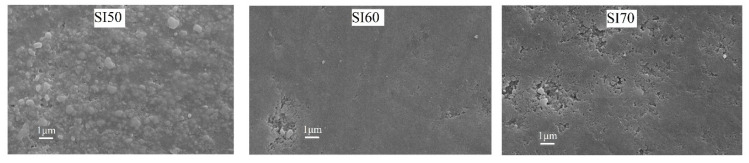
SEM micrographs of the polished surfaces of the calcinated samples SI50, SI60, and SI70. SEM magnification: 20 k×.

**Figure 6:**
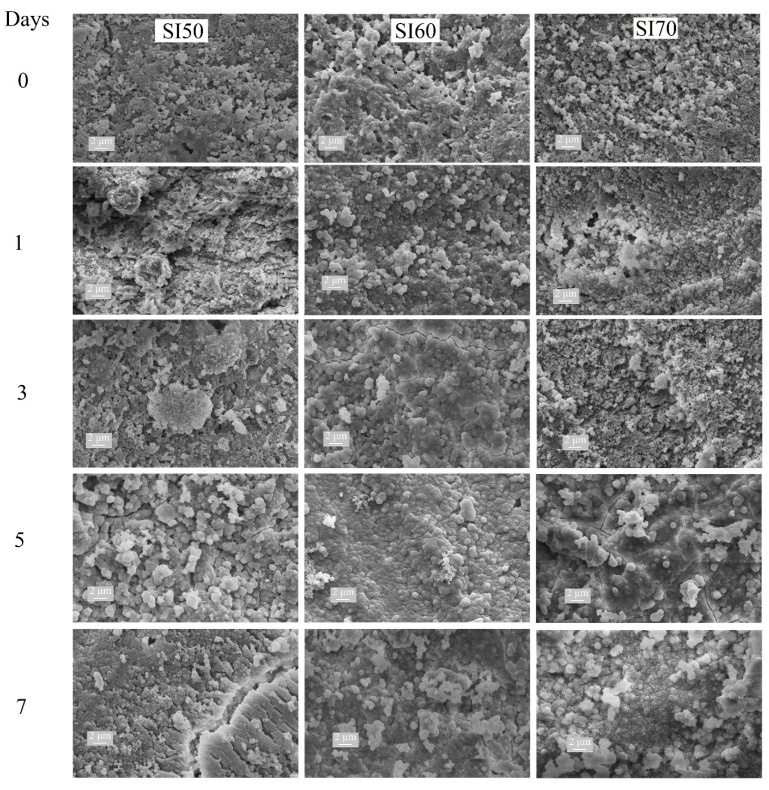
SEM micrographs of the samples SI50, SI60, and SI70 soaked in SBF for 0, 1, 3, and 5 days. SEM magnification: 10 k×.

Figure 5 depicts the microstructures of the SI50, SI60 and SI70 samples. A proper microstructural analysis helps to accurately discuss the mechanical properties. In the samples with 50% Si_3_N_4_, a coarse formation of grains can be visible, perhaps as a consequence of the higher concentration of the alkali activator and the formation of the cubic NaCaSiO_2_ phase. For the SI60 sample, finer grains with a homogenous morphology are evident, with no visible separation from the Si_3_N_4_ particles, confirming a good encapsulation of the material into the alkali-activated matrix. The SI70 sample exhibits a similar microstructure but with a visible increase in porosity. As observed, the formation of a compact sample corresponds to enough formation of the alkali-activated matrix, thereby improving the mechanical properties. This is confirmed by the measured relative densities found using the Archimedes method, reported in Table 2, in which the extent of the alkali-activation reaction is correlated with an increase in density. A better outlook is given by the micrographs of the unpolished surface, as those in the first row (day 0) of Figure 6, where the typical microstructure of a product of alkali activation is visible especially in the SI60 sample even after calcination, characterized by a glassy-like morphology. This is partly visible also in the SI50 sample, though in a lower amount, whereas the SI70 exhibits a totally different microstructure, with seemingly unreacted particles and the absence of a glassy-like morphology.

### 3.3. Bioactivity

The samples were tested in simulated body fluid (SBF) for 1, 3, 5, and 7 days after calcination. The changes in the surface, associated with the hydroxyapatite (HAP) phase, were evaluated using scanning electron microscopy (SEM). Figure 6 illustrates the evolution of surface deposition and the formation of HAP, typically characterized by a fine microstructure with a cauliflower-like shape. The sample containing 50% Si_3_N_4_ seems to exhibit very low or negligible growth of HAP, as the typical microstructure cannot be identified even after 7 days of SBF testing. On the contrary, the samples SI60 and SI70 seem to have an extensive production of HAP after 7 days, and even only after 5 days in the SI70 sample. Interestingly, the SEM images of the SI60 sample show a gradual transition of the surface morphology towards that of the HAP one, but the SI70 sample shows a sudden formation of HAP after 5 days, with copious precipitation of the same after 7 days. This anomaly further corroborates the relevance of the effect of the alkaline activator concentration in the formation of the alkali-activated matrix, even in terms of bioactivity. 

**Figure 7:**
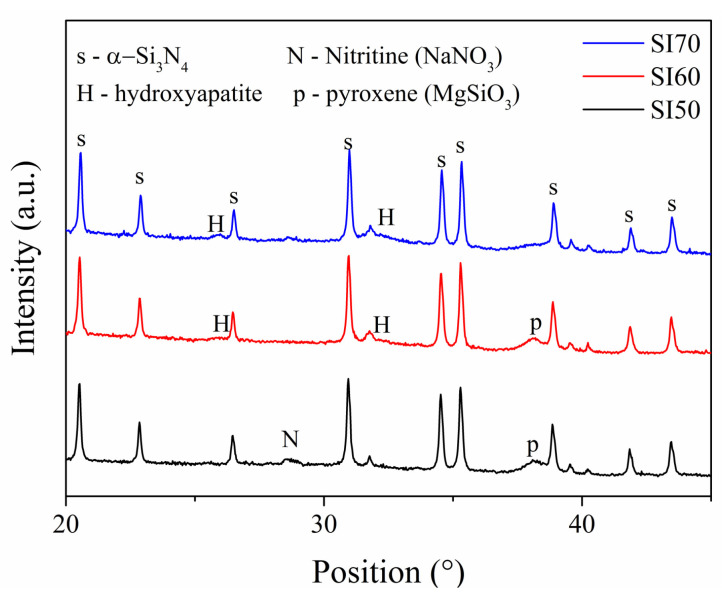
XRD patterns of the samples SI50, SI60 and SI70 after soaking them in SBF for 7 days.

X-ray diffraction of the samples after 7 days of SBF test was performed to confirm the production of the HAP, and the patterns are reported in Figure 7. The Rietveld refinements of the SI70 pattern of Figure 7 confirmed an extensive presence of HAP up to 30 wt%, in agreement with the cauliflower-like morphology found on the surface of this sample. The SI60 sample also exhibited a presence of HAP, but in a much lower amount (5 wt%), whereas in SI50, it was negligible (~1 wt%). These two samples, interestingly, showed the presence of other phases seemingly connected to MgSiO_3_, both monoclinic (pyroxen) and orthorhombic (enstatite) phases, the latter being much more prominent in the SI50 sample. The formation of these two phases is undesired and the causes are unknown. This means that the matrix formed in the SI70 sample, though not ideal from the alkali-activation standpoint, is easily dissolvable into SBF resulting in extensive production of HAP. In contrast, the samples synthesized with a stronger alkali activator produce a matrix evidently less reactive in SBF. Moreover, the XRD patterns of all three samples do not show the characteristic peaks of the ceramic combeite phase or cubic NaCaSiO_2_ phase, meaning that after 7 days, in the matrix, at least on the surface, these phases are entirely consumed by the SBF solution.

## 4. Discussion

The main counterintuitive result of this study comes from the samples with compositions at the extremes of the compositional range. Figure 1 demonstrates how different concentrations of the alkaline activator produce different compositions of the matrix, such as the production of a cubic NaCaSiO_2_ phase in the SI50 and SI70 samples. The presence of this phase indicates that a lower efficiency of alkali activation occurs in these compositions for two different reasons: in the SI50, the activator is so concentrated that NaOH is consumed in the formation of NaNO_3_, whereas in the SI70 sample, the molarity of the activator is not enough to completely dissolve the silica powder and to provide the appropriate stoichiometry for combeite formation. Indeed, the latter inefficiency is reflected in the Rietveld refinements of the calcinated samples, resulting in compositions that are inconsistent with the theoretical ones. As a consequence, this sample is shown to be less robust when mechanically tested (in all the tests, this sample always turned out to be the worst of the set, see Figure 3 and Figure 4). The low concentration of the alkaline activator produces a microstructure that is too porous with visible unreacted particles (see Figure 5 and Figure 6), as proved by the density calculation. However, this inefficiency is not only not a determining factor in the bioactivity test of Figure 6, but actually, the presence of the amorphous unreacted particles of silica increases the rate of element dissolution in the SBF solution thus involving a better bioactivity than the other samples. However, the observation that the bioactivity of this sample is triggered only after 5 days remains an unclear singularity. On the contrary, a well-formed ceramic matrix, such as that in the samples with a higher concentration of the alkaline activator, is less dissolvable in SBF and therefore less prone to reprecipitate HAP of the sample surface. However, the production of undesired phases during alkali activation, such as nitritine (Figure 1a), provokes the production of cracks after calcination which highly impairs the mechanical properties, especially of the samples SI50 and SI40. The SBF test of these samples gives a final hint which allows the completion of the picture so far exposed: the inefficiency of alkali-activation reaction is a deleterious factor for the samples with a higher content of matrix due to the extremely high concentration of the alkali activator, giving rise to a bioinactive matrix. When the optimal concentration is achieved (i.e., SI60 sample), the HAP is then produced in SBF after 7 days of soaking in the solution, and high mechanical properties are achieved. Interestingly, the lower concentration of alkali activator, which is responsible for the inadequate silica dissolution resulting in a drop in the mechanical properties, somehow produces a material that is decisively more bioactive. Finally, in this work, the SI60 sample is considered to be the best in the set of samples. 

Despite all the side effects, compared to other AAMs exhibiting σ_c_ values of approx. 50 MPa, our best result, that is from SI60, is slightly lower than the average (28 MPa), but the strength decisively improves after calcination, becoming up to 40% higher than a typical AAM, and displaying a five-fold increase in hardness, confirming that the calcination stage is a step that cannot be dismissed from the synthesis route [44,45,46]. However, when comparing them to bioactive ceramics and glass ceramics, our material presents lower mechanical properties, but they are not far from the target of 150 MPa of the theoretical bulk combeite material [47] and the cortical bones [48]. A better perspective on the quality of this material is provided by comparing it with biocements widely used in dentistry, such as glass ionomer cements (GICs) modified with bioglass particles for bioactivity [49,50]: our material proves to have values for the compressive strength in agreement with the average of the GICs and surface hardness and IFT values that are even higher, to the point of being even higher than those attained with other Si_3_N_4_-based composite ceramics [16]. We assume that these improved mechanical properties, especially the IFT, are due to a better incorporation of the silicon nitride powder in the matrix, following the surface functionalization of the silica layer on the particles’ surface. As far as the bioactivity is concerned, these materials exhibited a superior bioactivity response when compared to geopolymers and other AAMs, as usually 21 days are necessary to observe the same HAP precipitation [44,45]. Comparing the same properties with bioceramics, and especially with combeite or recrystallized bioglasses, our samples are still found to be better, as typically, ceramic materials show extensive formation of HAP after 14 days [51]. In future works, other strategies of alkali-activation procedures and paste settings will be attempted to improve the quality of the alkali-activated product, in particular producing a carbonated-free material by curing in a vacuum or inert atmosphere, thus avoiding the inefficiency of alkali-activation reaction by producing a preformed sodium silicate paste with additives to mix with the Si_3_N_4_ particles. In this way, we expect to be able to produce comparable samples irrespesctive of the matrix content, thus enabling us to conduct a systematic study of the proportions of matrix-Si_3_N_4_ content and, at the same time, to further improve the materials in terms of mechanical properties (reaching compressive strength values above 150 MPa) and bioactivity (observing deposition of HAP after fewer days of SBF testing).

## 5. Conclusions

This study has focused on the development of a bulk biocomposite formed of silicon nitride particles encapsulated in a bioactive matrix produced through the alkali-activation technique. This method was chosen for its simplicity and ability to obtain the required shape for the respective molds. In terms of mechanical properties, it was observed that increasing the matrix or silicon nitride content decreases the compressive strength in both alkali-activated and calcinated samples. The optimal value of strength was found in the composition with 60% silicon nitride content, with a micro-hardness of 1.61 GPa, an indentation fracture toughness of 0.34 MPa∙m^1/2^ and a compressive strength of up to 110 MPa. As expected, the mechanical strength values of the calcinated materials are in all cases well above the AAMs’ values. Thermal and XRD analyses showed that at 750 °C, the combeite phase was largely formed in this sample, leading to an increase in mechanical properties, but in other compositions, the formation of the cubic NaCaSiO_2_ phase was observed alongside combeite. This is a consequence of an inefficiency of dissolution during the alkali-activation method. The bioactivity test showed a positive response to simulated body fluid (SBF) for increasing amounts of Si_3_N_4_, because a stronger alkali activator produces a less bioactive matrix. The optimal sample, anyway, showed fairly good bioactivity, with an extensive production of hydroxyapatite after 7 days in SBF. It is, therefore, possible to develop bioactive materials with good mechanical properties using the alkali-activation technique. However, FTIR and XRD analysis revealed the formation of carbonates during the process. Therefore, the study and optimization of the alkali-activation process is essential to prevent carbonate formation and improve the efficiency of the method, and to do so, future studies will include a curing stage in vacuum or inert atmosphere and the ex situ preparation of a sodium silicate precursor for the synthesis of the matrix. 

## Figures and Tables

**Table 1:** Chemical composition for designing the slurry and the bulk of each sample.

Samples	Si_3_N_4_ (wt%)	Composition of the Matrix (wt%)				Molarity of the Alkaline Activator (M)
		SiO_2_	Ca(OH)_2_	MgO	NaOH	
**SI40**	40.00	23.25	16.75	3.10	16.90	30.72
**SI50**	50.00	19.38	13.95	2.58	14.09	25.60
**SI60**	60.00	15.50	11.16	2.07	11.26	20.48
**SI65**	65.00	13.56	9.77	1.82	9.86	17.92
**SI70**	70.00	11.63	8.37	1.55	8.45	15.36
**SI80**	80.00	7.75	5.58	1.03	5.63	10.24

**Table 2:** Calculated relative density measurements with their respective errors according to the Archimedes method.

Composition	SI50	SI60	SI70
Relative density (%)	75.67 ± 0.21	71.74 ± 0.61	68.31 ± 0.07

## Data Availability

Data are contained within the article.
